# Motor–Speech Performance in Very Old Speakers: Associations With Physio‐Anatomical and Cognitive‐Linguistic Factors

**DOI:** 10.1111/1460-6984.70137

**Published:** 2025-10-23

**Authors:** Sonja Alantie, Tanja Makkonen, Kati Renvall

**Affiliations:** ^1^ Department of Psychology and Speech–Language Pathology University of Turku Turku Finland; ^2^ Speech–Language Pathology Tampere University Hospital Tampere Finland

**Keywords:** articulation rate, motor–speech performance, oral DDK, semi‐spontaneous narrative, speech rate, very old age

## Abstract

**Background:**

Motor–speech skills slow down with age, but health care professionals lack normative data, especially on the vastly growing population of very old (VO) speakers. The execution of different motor–speech tasks requires both fine‐motoric and cognitive abilities.

**Aims:**

To study the performance on oral diadochokinetic (DDK) rate and narrative speech tempo in typically ageing 80–100‐year‐old speakers and to investigate whether they are predicted by age, dentition, hearing, cognitive status, language skills or educational level.

**Methods:**

This cross‐sectional study comprises 50 typically ageing VO Finnish speakers. Their motor–speech performance was evaluated by alternating motion rate (AMR) syllables /pa/, /ta/ and /ka/ and sequential motion rate (SMR) syllable sequence /pataka/ and two speech tempo parameters (speaking and articulation rate) in semi‐spontaneous narrative. The association between task performance and background variables was studied by multiple linear regression analysis.

**Results:**

The VO speakers’ normative performance in DDK, speaking and articulation rates was predicted by physio‐anatomical and cognitive‐linguistic factors. Older age within the 80–100‐year range was associated only with slower execution of the SMR task. Wearing dentures predicted slower tempo in the AMR tasks and articulation rate. The highest educational level predicted slower tempo in the AMR tasks. Good language skills were positively associated with motor‐speech performance: Better phonemic fluency predicted faster AMR /pa/ and SMR /pataka/, and a higher Western Aphasia Battery Aphasia Quotient predicted a faster speaking rate.

**Conclusions:**

The VO speakers had relatively well‐preserved motor–speech skills. Consistent with previous studies, the mean DDK, speaking and articulation rates were, nevertheless, slower in the VO speakers than in younger speakers in prior research. As a novel finding, SMR was slower than AMR in the VO speakers, which deviates from the trend observed in Finnish adults and younger elderly. This study suggests that natural teeth, younger age and good language skills safeguard the motor–speech skills from slowing down. However, it seems characteristic for the most highly educated VO speakers to perform slower than peers in the AMR tasks.

**Contribution:**

The results of this study will help to identify the manifestations of typical ageing. They also give insight into the life‐long evolution of speech skills and into the relationship between the motoric and linguistic facets of speech.

**WHAT THIS PAPER ADDS:**

*What is already known on the subject*

- Motor–speech performance slows down with ageing because of physio‐anatomical and cognitive‐linguistic changes. Speech tempo is, thus, a sensitive biomarker for neurological alterations.

*What this study adds to existing knowledge*

- Formerly missing data on very old (VO) speakers’ typical motor–speech skills and their predictors. Within‐group differences are promoted by age, dentition, language skills and educational level.

*What are the clinical implications of this work?*

- DDK, speaking and articulation rates are suitable means for clinical motor–speech assessment even in the oldest speakers. In addition to chronological age, speech–language pathologists should consider a range of individual physio‐anatomical and cognitive‐linguistic factors that contribute to speech performance. We recommend conducting AMR tasks /pa/, /ta/ and /ka/ together with an SMR /pataka/ task and comparing the results with culturally valid age‐specific norms (if available), as their atypical relationship may indicate neuropathology.

## 1 Introduction

Motor speech performance has been found to slow down with age (Bóna 2014; Jacewicz et al. 2009; Kent et al. 2022; Verhoeven et al. 2004). However, the underlying factors contributing to the decline are not yet fully understood (Mefferd and Corder 2014). On one hand, the slowing down has been attributed to age‐related changes in the anatomy and physiology of the speech and breathing organs and in the changes of fine motor‐control and sensory feedback (Bennet et al. 2007; Chisolm et al. 2003; Mefferd and Corder 2014; Tremblay et al. 2017). On the other hand, cognitive‐linguistic factors such as changes in executive functions and lexical access have been noted to affect the tempo of speech with age (Hooper and Cralidis 2009; Martínez‐Nicolás et al. 2022; Tremblay et al. 2017). The population worldwide is ageing, and so is the speech–language pathologists’ clientele. Nevertheless, little is known about how very old age affects motor speech performance or which individual factors influence the skills.

Motor–speech functions are frequently assessed in speech–language pathology (SLP) by oral diadochokinetic (DDK) rate, including alternating motion rate (AMR), which is the speed of repeating a single syllable (e.g., *pa pa pa…*), and sequential motion rate (SMR), which is the speed of repeating a sequence of different syllables or a non‐word (e.g., *pataka pataka…*) (Kent et al. 2022). Oral DDK is a simple yet informative way to measure articulatory mobility, articulatory agility, motor speed, motor coordination and sequencing. Other often utilized tempo measures include speaking and articulation rates (Niimi and Nishio 2001). They both demonstrate the pace at which a stretch of connected speech—such as spontaneous or elicited narrative—is produced, with the distinction that speaking rate includes pauses while articulation time excludes them (Bóna 2014; Niimi and Nishio 2001; Yunusova et al. 2016).

For this study, we apply the terminology used, for example, by Forman et al. (1992) referring to young‐old (60 to 69 years), middle‐old (70 to 79 years), and very old (henceforth VO) (80 years and older) individuals. We use the term *old* as an umbrella term referring to all the above‐mentioned age groups. There are few studies that investigate DDK rates and speaking and articulation rates related to normal ageing in the young‐old or middle‐old and even fewer that include very old speakers (Bóna et al. 2014; Hooper and Cralidis 2009; Kent et al. 2022; Pierce et al. 2013). We will next provide an overview of the skills required in motor–speech tasks and what is already known about the effect of old age itself and other background variables, namely dentition, hearing, cognition, language skills and education in relation to motor–speech functions.

### 1.1 The Nature of Motor–Speech Tasks

DDK, speaking and articulation rates are all recognized as functional biomarkers for neurological disorders, because of their sensitivity to neural change (Duffy 2013; Kent et al. 2022; Rodgers et al. 2013; Yunusova et al. 2016). The tasks require functional motoric skills and at least some degree of cognitive‐linguistic skills. This should give them the potential to detect also more subtle neurological alterations, such as the ones occurring in ageing.

Typically, DDK, speaking and articulation rates have been used to assess the sensory‐muscular and coordinative dimension of speech, while the cognitive‐linguistic procedural skills involved have received less attention. Previous literature has not analysed the precise motoric and cognitive skills required for producing AMR, SMR and narrative speech nor how the skill requirements compare to each other. According to Duffy (2013, 50–51) speech is a multifaceted system with five levels: (1) *conceptualisation*, (2) *linguistic planning*, (3) *motor planning or programming*, (4) *performance* and (5) *feedback*. Based on this and our clinical experience, we suggest that AMR, SMR and narrative pose different requirements on the speaker as follows: Narration, as a task including both the generation of semantic content and the articulatory production of speech, requires all levels from conceptualisation to feedback. SMR, as a repetition task of a nonword with a three‐syllable‐sequence containing familiar phonemes but an unfamiliar combination, requires a process from linguistic planning to feedback. AMR, as a repetition task of a familiar monosyllable, requires the same processes as SMR but involves less linguistic and motor planning.

We elaborate on the motoric and cognitive‐linguistic requirements further (see Duffy 2013, 50–51, 80–81). Motorically, the trisyllabic SMR is very demanding. A speaker needs motoric planning and control skills to alternate between three syllables of different articulation places and manners, formulating an unfamiliar syllable sequence while upholding a rapid production speed and temporal coordination. Narrative speech is also motorically quite demanding, as it requires the production of whole intelligible sentences, but it also provides more liberty of using habitual speech tempo and planning the motoric composition of words through vocabular choices. AMR, again, is motorically the simplest; a familiar monosyllable is produced by pursuing maximal speed and adequate articulatory precision with temporal coordination.

From a cognitive‐linguistic perspective, we claim that narrative speech is the most demanding, followed by SMR and AMR, respectively (see Duffy 2013, 50–51, 80–81). In short, narrative speech requires cognitively generated thoughts and a desire to express them in order to achieve a communicative goal. It then requires interactive semantic and morpho‐syntactic processing, finally taking phonological form. This language planning demands attention, retrieval and working memory processes. The SMR task also requires attention and working memory skills, as the speaker needs to recognise, rapidly learn and retain a novel trisyllabic non‐word and monitor its production in fast repetition. The AMR task requires at least attention; a speaker must recognise and retain a familiar monosyllable and monitor its production in fast repetition. Naturally, all these tasks also require the comprehension of the task instructions to begin with.

### 1.2 The Effect of Ageing on Motor–Speech Performance

The general view of ageing and oral DDK shows that the rates increase until early or middle adulthood and then decline with advanced age (Kent et al. 2022). More accurately, the decline in DDK rate is reported to occur after 60 years of age (Iyota et al. 2020). The decline may either plateau or be very slow between the ages of 65 and 86 (Kikutani et al. 2009; Pierce et al. 2013). It is suspected that further slowing might occur again after 85 years of age (Pierce et al. 2013), but there is not enough research to support this hypothesis (cf. Kent et al. 2022). In Finnish, the DDK rates in healthy adults and old speakers have been studied only in an unpublished master's thesis by Nevala (2014). The comparison between 18 and 40 (*n* = 44) and 70–75‐year‐olds (*n* = 30) showed that old speakers were statistically slower than adult speakers in AMR /pa/, /ta/, /ka/ and SMR /pataka/.

Similarly, speaking and articulation rates decline with ageing (Amir 2016; Bóna et al. 2014; Duchin and Mysak 1987; Jacewicz et al. 2009; Quene 2008; Verhoeven et al. 2004). The critical cutoff age for decline in speaking and articulation rates is difficult to determine by the existing research. For example, Duchin and Mysak (1987) and Verhoeven et al. (2004) found significant differences already between the ages of 40 and 45 years. Most studies, however, demonstrate a statistically significant difference in speaking and articulation rates between under 40 years and over 60 years of age (e.g., Amir 2016; Bóna et al. 2014). Bóna et al. (2014) studied speaking and articulation rates in younger (21–32 years) and older (66–90 years) speakers who completed four different speech tasks: spontaneous narrative, narrative recall, reading aloud and conversation. Old speakers had slower articulation rates in all tasks and slower speaking rates in all except narrative recall. Similarly, Duchin and Mysak (1987) demonstrated age‐related decline in speaking rates in conversation, reading aloud and picture description, when they studied male speakers between the ages of 21 and 91 years. These studies indicate a systematic age‐related slowing in speech tempo across different speech acts and registers. In Finnish, normative speaking and articulation rates in semi‐spontaneous narrative can only be found for a population of 18–89‐year‐olds (*N* = 70) used as a control group for speakers with neurogenic stuttering (Penttilä et al. 2018).

### 1.3 The Effect of Dentition and Hearing on Motor–Speech Performance

When it comes to age‐related physio‐anatomical changes, the loss of natural teeth is one factor influencing speech. Firstly, wearing dentures alters articulation and may slow down speech. Dentures mechanically change the space in the oral cavity and affect mandible and tongue movements required for making speech sounds (Freitas de Souza et al. 2007; Kikutani et al. 2009). Dentures also often alter oral proprioception and intraoral sensory perception, affecting the planning, control and feedback of speech movements (Ghi and McGivney 1979; Hermes et al. 2018). Secondly, poor oral health may affect speech regardless of whether a person uses dentures to replace missing teeth. A growing body of research indicates a connection between poor oral health and cognitive decline (Fang et al. 2018). As speech is affected by cognition (Martínez‐Nicolás et al. 2022), there might be an indirect connection between tooth loss and a slower motor‐speech performance.

Furthermore, oral health has an association with systemic or physical health (Kane 2017). Periodontal disease, which is a common cause of tooth loss, is associated, for example, with atherosclerotic vascular and pulmonary diseases and metabolic disorders. There is support for the concept that persons with poorer physical health have slower speaking rates (Duchin and Mysak 1987). Tooth loss and periodontal disease are also associated with frailty (Hakeem et al. 2021), which is a medical condition marked by a decline in physical strength, endurance and function, but also with diverse measures of executive‐cognitive functions and oral frailty (Dos Santos Nogueira et al. 2023). Therefore, tooth loss and oral health can be associated with poorer motor–speech functions in at least three ways: (1) by causing oral sensory‐mechanical changes (2) by connecting to neuro‐cognitive functions and (3) by representing the overall physical health status.

Based on the following studies, dentition may even be a more important predictor of motor–speech functions than old age itself. Kikutani et al. (2009) studied oral DDK rate in three age groups: 65–69‐year‐olds, 70–74‐year‐olds and 80‐year‐olds or older. The participants were further divided into two groups based on their dental status: participants with naturally adequate dentition and participants wearing dentures. Older age was a significant predictor of declined DDK rate only in denture wearers. Kikutani et al. (2009) suggested this could be explained by the mechanical alteration dentures inflict on motor‐speech production. Hara et al. (2017) investigated the association between oral conditions and DDK rate in elderly Japanese (> 65 years of age). They found that over a 1‐year period over 20% of the elderly had a decrease in their DDK rate. Their results suggested that the number of untreated teeth was closely related to the decline in the DDK rate.

In most individuals, ageing entails some level of sensory impairment, for example, hearing loss, also called presbyacusis (Chisolm et al. 2003; Hazan et al. 2018). There is little relevant research on how presbyacusis affects motor–speech functions in terms of articulatory control or speech tempo. In the study by Hazan et al. (2018), older speakers with hearing loss as well as speakers without hearing loss exhibited slower articulation rates when speaking in challenging listening conditions or to a listener with hearing loss. In adults with acquired hearing loss, inadequate auditory feedback during speech may result in degradation of motor–speech performance (Duffy 2013). Therefore, we hypothesise that poor hearing could also result in slower motor–speech performance in typically ageing speakers either because they aim to ensure speech clarity for both them and the listener, or because utilizing auditory feedback may be more difficult during rapid speech.

### 1.4 The Effect of Cognitive and Language Abilities on Motor–Speech Performance

The interrelation between motor–speech and cognitive‐linguistic skills is complex. Both the cognitive abilities of the speaker and the cognitive requirements of the speech act affect the speech performance (e.g., Dromey and Benson 2003; MacPherson 2019). For example, according to Dromey and Benson (2003), cognitive‐linguistic strain of a task induces variability in its motoric execution. They conclude that neural resources allocated for communication shift to different aspects of it, for example, to articulation or to semantic content, depending on the demands of the situation. Ageing itself causes cognitive changes, including decline in executive function, such as processing speed, working memory, attention and inhibitory control (Cahana‐Amitay and Albert 2014). Therefore, older speakers tend to be more sensitive to situational strain, like a study by MacPherson (2019) indicates. According to it, the speech stability and timing in older (68–78 years) speakers was more affected by the task‐related cognitive load than in the younger speakers (22–32 years). Furthermore, among old speakers, dementing illnesses are a common source for variation in cognition and speech‐motor performance. In the study by Nagumo et al. (2020), 65–96‐year‐old healthy speakers could be differentiated from speakers with mild to severe cognitive impairments by their speech. For example, DDK rate was reduced in individuals with cognitive impairment. In addition to Nagumo et al. (2020), we have not been able to discover other studies reporting the relationship between the primary cognitive abilities of typically ageing VO speakers and their motor–speech performance. In fact, many previous studies examining old speakers have not screened for their cognitive skills.

Age‐related cognitive changes cause progressive deterioration in language abilities, which may alter motor–speech skills (Cahana‐Amitay and Albert 2014). Linguistic changes involve mainly problems with lexical access and sentence processing. Word retrieval and speech planning difficulties may manifest, for example, as more frequent or longer pauses in connected speech (Spieler and Griffin 2006), which again result in a slower speaking rate (Bóna 2014). In the study by Bóna (2014), 66–99‐year‐olds, and in the study by Martins and Andrade (2011), over 80‐year‐old speakers produced pauses more frequently than younger speakers. However, the influence of word retrieval or speech planning difficulties on speech tempo is not straightforward. Instead of silent pauses, older speakers may use more circumlocutions or filler words (e.g., Mortensen et al. 2006) as a compensatory strategy for word‐finding deficits. To our knowledge, there are no studies investigating the relationship between language skills and motor–speech performance in VO populations.

The relationship between educational history and motor–speech skills is still sundry. In neurosciences, the level of one's education is first and foremost regarded as a representative of a person's cognitive abilities and intelligence, but it also reflects personal characteristics and environmental circumstances (see Alantie et al. 2022). In many modern societies, the quality and the length of formal education acquired by the old differ from the level acquired by the young and working‐age populations. Therefore, knowledge about the effects of education in adult speakers may not be applicable to the older generations. Nevertheless, it has been suggested that education, together with many other social background variables, is a source of between‐speaker variation of speech tempo in adults (Jacewicz et al. 2009). Education is, thus, recognised as having a role in how we speak during our lifespan. To our knowledge, there are no studies investigating the association between educational background and motor–speech skills in an old population.

### 1.5 Research Questions

The rationale of this study is to provide data on the typical motor‐speech performance of the VO speakers and to identify contributing background factors. The results of this study will help speech–language pathologists and other health care professionals to identify the characteristics of typical ageing. This study addresses the questions:

- What are typical DDK, speaking and articulation rates in very old (VO) speakers?
- Are the speakers’ physio‐anatomical factors (age, dentition and hearing) and cognitive‐linguistic abilities (cognitive status, language skills and educational level) associated with the motor–speech task performance?

## 2 Methods

### 2.1 Participants

The inclusion criteria for this cross‐sectional study were (a) age of at least 80 years, (b) native speaker of Finnish, (c) no diagnosed or suspected dementing illness, (d) no diagnosed speech or language disabilities and (e) a Mini‐Mental State Examination test (MMSE) score of 24–30 (for a Finnish version see, Hänninen et al. 2010). Volunteers were recruited through open advertisements and visits to local groups for the elderly and private senior homes and by word‐of‐mouth. The study comprises altogether 50 community‐dwelling participants (females *n* = 41, males *n* = 9), including one person with myasthenia gravis with a good medication balance. Educational level, hearing and dental status were obtained by self‐reports. Over half of the subjects (*n* = 28) wore complete or partial dentures. Poor hearing was reported by 24 out of 50 participants, and 14 of them used a hearing aid. The rest considered their hearing adequate. Demographic variables of the participants are presented in Table 1. Written informed consent was provided by all the participants. Ethical approval for this study was obtained from the Regional Ethics Committee of the Tampere Human Sciences.

**TABLE 1:** Demographic and language variables of the very old speakers (*N* = 50).

Age (years)	MMSE	Educational level	Dentition	Hearing	WAB AQ	Semantic fluency	Phonemic fluency
Range 80–100 *M* = 84.8 SD = 4.1	Range 24–30 *M* = 27.6 SD = 1.8	Primary, *n* = 18 Secondary, *n* = 20 Tertiary, *n* = 12	Natural, *n* = 22 Dentures, *n* = 28	Adequate, *n* = 26 Poor, *n* = 24	Range 84.9–99.7 *M =* 95.7 SD *=* 3.4	Range 8–38 *M =* 21.5 SD *=* 2.8	Range 2–26 *M =* 13.8 SD *=* 5.6

Abbreviations: M = mean value, MMSE = Mini‐Mental State Examination, Primary = primary education or lower secondary education, SD = standard deviation, Secondary = upper‐secondary education or post‐secondary non‐tertiary education, Tertiary = Bachelor's or equivalent level or Master's or equivalent level education, WAB AQ = Western Aphasia Battery Aphasia Quotient.

### 2.2 Language Performance

We used three common linguistic tests as predictor variables. We conducted the Finnish version of the Western Aphasia Battery (Kertesz et al. 2005), from which we utilized the Aphasia Quotient (AQ, max 100) as the summary value of a speaker's general competence in oral expression and auditory comprehension. To assess time‐pressured lexical access and cognitive executive functions (see Alantie et al. 2022), we conducted a semantic animal category task and a phonemic word‐fluency task with the initial letter /k/. Fluency tasks entailed the generation of as many words as possible belonging to the given category within a minute. Test scores are presented in Table 1. For details on administering and scoring the language tasks and for the Finnish normative reference data see, Appendix B.

### 2.3 DDK Rates

To measure maximal motor speed, articulatory mobility, coordination and sequencing, we conducted the oral DDK utilizing AMR monosyllables /pa/, /ta/, and /ka/ and the SMR trisyllabic sequence /pataka/, which is also a non‐word. The participants were asked to produce AMR and SMR as fast as they could for at least 5 s. The examiner modelled the tasks, and the participants were encouraged to practise uttering the SMR sequence before the test round. The examiner gave the participants an auditory cue to start and stop the task. The tasks were audio recorded with a Zoom recorder and a headset microphone. The PRAAT program (Boersma and Weenink 2024) was used to extract 5‐s samples and to verify and count syllables acoustically and visually. The continuous 5‐s sample was extracted from the speaker's steadiest performance, for example, omitting some start‐up difficulty. The DDK rates were counted by the number of accurately produced and correctly positioned syllables and expressed as syllables per second (SPS).

### 2.4 Speaking and Articulation Rates

To assess the motor speech tempo in connected speech, the participants were asked to generate a story from a wordless, nine‐picture cartoon strip (The Scarecrow Cartoon “Fugleskraemsel går amok” by Henning Dahl Mikkelsen, see Korpijaakko‐Huuhka, 2003), which has also been used in other Finnish studies investigating motor–speech production in adults with and without neurological disorders (Makkonen et al. 2018; Penttilä et al. 2018). We chose to use a semi‐spontaneous narrative, as it is cognitively more demanding than reading, and unlike a fully spontaneous narrative, it poses similar cognitive‐linguistic requirements for all the speakers. The participants were asked to tell the story in their own words with all the preparation time they needed. The stories were audio recorded with a Zoom recorder and a headset microphone and annotated orthographically with PRAAT. Pauses include silent and filled pauses of 200 milliseconds (ms) or longer (Niimi and Nishio 2001). Filled pauses in this study comprise hesitation sounds (/öö/, ‘er,’ /mm/ ‘um’) and non‐verbal sounds like coughing, laughter, or audible inhalation. Filler words (niinku ‘like,’ tuota noin ‘so’) or interjections (juu ‘okay’) were not included in filled pauses. To study the number of syllables, we created a semi‐automated system using mechanical annotation and automated counting by a corpus tool. Spoken Finnish differs from its written form and is rich in dialectal varieties, which made any available fully automated syllable counting tools error‐prone. The corpus tool used in this study was coded for the purpose of this study. Speaking rate was measured as the number of syllables used within the narration and divided by the total length of the speech act (Niimi and Nishio 2001). Articulation rate was measured as the number of syllables used within the narration and divided by its length without the duration of pauses. Speaking and articulation rates are expressed in syllables per second (SPS).

### 2.5 Procedure

Participants were studied by the first author either in their own home or at the university, according to their preference. The protocol entailed (1) history of educational, social and health status, (2) Boston Naming Test (Laine et al. 1997), (3) semi‐spontaneous narrative, (4) DDK AMR a) /pa/, b) /ta/ and c) /ka/ and SMR /pataka/, (5) WAB (Kertesz et al. 2005), semantic fluency and phonemic fluency, (6) MMSE (Hänninen et al. 2010) and (7) spontaneous narrative. Tasks (2) and (7) are not included in this study. The order of administration was constant across participants, and the protocol was executed within a single session, taking altogether 1–2 hours. Sessions were audio‐ and video‐recorded for scoring, transcription and analysis.

### 2.6 Statistical Analyses

To address our aim in providing normative data on the VO speakers performance in DDK and speaking and articulation rates, we counted the ranges, means and standard deviations of the tasks. To investigate the contribution of age, dentition, hearing, MMSE, WAB AQ, semantic fluency, phonemic fluency and educational level in predicting the performance in DDK, speaking and articulation rates, a multiple linear regression was conducted. A backward elimination approach, guided by the Akaike Information Criterion (AIC), was applied by initially including all variables in the model and sequentially removing those that were non‐significant. The AIC balances model fit with parsimony, considering both the accuracy of the fit and the number of parameters used.

Outliers in continuous predictors were winsorized to within 2.5 standard deviations of the mean for each variable to minimize their impact on the mean score. The dataset contained minimal missing data: only two values were missing among the outcome variables, and no missing data existed among the predictor variables. Given this, listwise deletion was applied to handle missing data without impacting the validity of the analyses. Regression assumptions were verified by assessing normality and homoscedasticity through visual inspection of residual plots. Multicollinearity was evaluated using the adjusted generalized variance inflation factor (aGVIF); all values were below 2, suggesting low multicollinearity. Education levels were coded from 1 to 3, representing primary, secondary, and tertiary education levels, respectively. All statistical analyses were performed using R statistical software (v4.3.0; R Core Team 2023).

## 3 Results

### 3.1 DDK Rates

The AMR and SMR rates in VO speakers are presented in Table 2. The mean speed order from fastest to slowest in the VO speakers’ DDK rates was as follows: /pa/, /ta/, /ka, and /pataka/. Individual variation was the greatest in the SMR /pataka/ sequence. Only two individuals were unable to complete a single DDK task.

**TABLE 2:** Oral diadochokinetic (DDK) rates in the very old speakers.

Variable	/pa/ (SPS), *N* = 49	/ta/ (SPS), *N* = 50	/ka/ (SPS), *N* = 50	/pataka/ (SPS), *N* = 49
*M*/SD	6.2/0.6	6/0.7	5.6/0.7	5.5/0.9
Range	4.6–7.6	4.6–7.4	4–7	3–7.8

Abbreviations: *M* = mean value, SD = standard deviation, SPS = syllables per second.

### 3.2 Speaking and Articulation Rates

Narrative tempo variables in VO speakers are presented in Table 3. There was notable between‐speaker variation in the narrative duration, total pause duration and in the number of syllables produced. The articulation rate was faster and more varied than the speaking rate.

**TABLE 3:** Duration, pauses and speech tempo in the cartoon strip narrations of the very old speakers (*N* = 50).

Variable	Narrative duration (s)	Number of syllables	Total pause time (s)	Speaking rate (SPS)	Articulation rate (SPS)
*M*/SD	70.8/40.3	217.5/116.2	24.6/17.3	3.2/0.7	4.8/0.7
Range	30.9–259.9	95–809	6.4–90.4	1.9–4.6	3.7–6.8

Abbreviations: *M* = mean value, s = second, SD = standard deviation, SPS = syllables per second.

### 3.3 Background Variable Association With DDK, Speaking and Articulation Rates

A statistical multivariable analysis was performed to examine the contribution of age, dentition, hearing, cognitive screening status, language skills and educational level on motor–speech tasks. For the linear regression models for DDK, speaking and articulation rates, see Table 4. Correlations and *p* values of correlation of all the predictor and outcome variables are visible in Appendix A. The results show that hearing, MMSE or semantic fluency did not predict performance in motor–speech tasks. Age, dentition, WAB AQ, phonemic fluency and education, instead, all predicted the speed of production in either one or several tasks. Older age was associated with slower SMR /pataka/ (*p* = 0.033). Wearing dentures, in contrast to having adequate natural teeth, predicted statistically significantly slower performance in AMR /pa/ (*p* = 0.015), /ta/ (*p* = 0.031) and /ka/ (*p* = 0.050) rates as well as in narrative articulation rate (*p* = 0.028). Higher WAB AQ was associated with faster narrative speaking rate (*p =* 0.018), and better phonemic fluency was associated with faster AMR /pa/ (*p* = 0.003) and SMR /pataka/ (*p* = 0.002) production. The highest educational level predicted slower performance on AMR /pa/ (*p* = 0.003), /ta/ (*p* = 0.022) and /ka/ (*p* = 0.041) rates.

**TABLE 4:** Linear regression models for DDK, speaking and articulation rates and their predictors.

				Model metrics					
Variable	Beta	95% CI	*p* value	*R* ^2^	Adj *R* ^2^	Statistic	*p* value	AIC	No. Obs.
**AMR /pa/**				0.343	0.283	5.74	< 0.001	82.5	49
Education									
Secondary	−0.12	−0.49, 0.25	0.5						
Tertiary	−0.69	−1.1, −0.25	0.003**						
Dentition	−0.41	−0.73, −0.08	0.015*						
Phonemic fluency	0.05	0.02, 0.08	0.003**						
**AMR /ta/**				0.269	0.167	2.64	0.029	102	50
Education									
Secondary	−0.1	−0.54, 0.33	0.6						
Tertiary	−0.62	−1.2, −0.10	0.022*						
Dentition	−0.43	−0.83, −0.04	0.031*						
WAB AQ	0.07	−0.01, 0.14	0.078						
Phonemic fluency	0.03	−0.01, 0.07	0.1						
Semantic fluency	−0.03	−0.07, 0.01	0.2						
**AMR /ka/**				0.172	0.098	2.33	0.07	104	50
Education									
Secondary	−0.08	−0.52, 0.36	0.7						
Tertiary	−0.56	−1.1, −0.02	0.041*						
Dentition	−0.39	−0.78, 0.00	0.05*						
Phonemic fluency	0.03	−0.01, 0.06	0.12						
**SMR /pataka/**				0.268	0.237	8.44	< 0.001	124	49
Age	−0.07	−0.14, −0.01	0.033*						
Phonemic fluency	0.07	0.03, 0.11	0.002**						
**Speaking rate**				0.259	0.227	8.21	< 0.001	91.2	50
WAB AQ	0.07	0.01, 0.13	0.018*						
Phonemic fluency	0.03	0.00, 0.06	0.087						
**Articulation rate**				0.137	0.1	3.73	0.031	102	50
Hearing	−0.29	−0.65, 0.07	0.11						
Dentition	−0.41	−0.78, −0.05	0.028*						

*Note*: Reference levels: Education (primary), Dentition (natural teeth), Hearing (adequate).

Abbreviations: Adj *R* ^2^ = adjusted coefficient of determination, AIC = Akaike information criterion, CI = confidence interval, *R* ^2^ = coefficient of determination. Statistical significance: *p < 0.05, **p < 0.01.

## 4 Discussion

In this study we investigated the motor‐speech performance in typically aged very old (VO) speakers using oral DDK rates (including AMR /pa/, /ta/ and /ka/ and SMR /pataka/) and narrative speaking and articulation rates. We also studied whether physio‐anatomical factors (age, dentition and hearing) and cognitive‐linguistic abilities (cognitive status by MMSE, language skills by WAB AQ, semantic and phonemic fluency, and educational level) predicted the task performance.

### 4.1 How Did the Very Old Speakers Perform in DDK?

Based on the normative data on this study, VO speakers performed generally well in the DDK tasks. However, if the mean DDK rates in VO speakers are compared with the rates in Finnish adult (18–40 years) and middle‐old (70–75 years) females in the study by Nevala (2014), the VO speakers were consistently somewhat slower (e.g., AMR /pa/ SPS in adults = 6.9 ± 0.7, in the middle‐old = 6.6 ± 0.6 and in the VO = 6.2 ± 0.6). Relevant literature for DDK rates in VO speakers is scarce (Kent et al. 2022), but for example, in Japanese, which has a clear syllable structure like Finnish, old speakers also show the gradual slowing down in DDK between the ages of 40 and 87 years (Iyota et al. 2020). In the VO speakers of our study, individual variation was the greatest in the SMR /pataka/ task, which was also clinically the slowest of their DDK rates. In contrast, in the middle‐olds (70–75 years), /pataka/ was the second slowest, and in the adults, it was the fastest of their DDK rates (Nevala 2014). This implies that either the motoric or cognitive‐linguistic requirements or the combination of both in SMR /pataka/ become more laboured as ageing advances. It has been suggested that especially the articulatory movements of the tongue might become less controlled by ageing (Hermes et al. 2018). This could show in the production of /pataka/ with two different lingual sounds (apical /t/ and dorsal /k/).

### 4.2 How Was the Very Old Speaker's Speech Tempo?

Speaking and articulation rates in this study were marked by individual variation, with articulation rate being more varied than speaking rate. Comparison to another, mainly younger Finnish sample (*N* = 70, range 18–89 years) (Penttilä et al. 2018) suggests that age‐related slowing down is present in both speaking and articulation rates in semi‐spontaneous narrative (18–89 years speaking rate = 3.8 SPS, articulation rate = 5.6 SPS and VO speaking rate = 3.2 SPS, articulation rate 4.8 SPS). This finding conforms to other studies (Amir 2016; Bóna et al. 2014; Duchin and Mysak 1987; Jacewicz et al. 2009; Quene 2008; Verhoeven et al. 2004). According to Quene (2008), one possible explanation for the slower speech tempo is the older speakers’ tendency to use shorter phrases, which are generally produced at a slower pace than long phrases. We did not analyse utterance length, but this could be a subject of further interest.

### 4.3 Individual Factors in Motor–Speech Performance

The regression analysis on background variables revealed new and interesting information. Motor–speech performance was affected by physio‐anatomical as well as cognitive‐linguistic factors. Age itself, however, predicted only one of the tasks: SMR. This may be explained by the relatively narrow age range of this study. The possible critical cutoff‐age for change in many motor–speech functions may well have occurred earlier than in very old age, as some studies imply (e.g., Amir 2016; Bóna et al. 2014; Iyota et al. 2020). Given its motoric and cognitive‐linguistic complexity, the SMR task was, however, sensitive enough to reveal age‐related differences, even within the limited age range and sample size in this study. Additional slowing down can, thus, be detected still somewhere from 80 years onwards, at least in demanding motor–speech tasks.

The main physio‐anatomical contributor to motor–speech tasks was dentition. Wearing complete or partial dentures versus having natural teeth predicted statistically significantly slower performance in all AMR rates as well as in articulation rate. Although dental health has a connection with cognitive abilities, in our study dentition is more likely to have a sensory‐kinetic influence on the motor–speech tasks. AMR is a cognitively easy task. It enables the capitalisation of maximal motoric mobility, agility, speed and coordination, because speech movements are not decelerated by high cognitive processing requirements. As a test variable, articulation rate is also motorically emphasised; it is stripped from pauses that may entail cognitive processing (pauses of at least 200 msec have been omitted) (Niimi and Nishio 2001). Dentures can hamper the maximal motor–speech performance and, thus, differentiate speakers from each other for the following four reasons. First, wearing dentures changes the oral space, affecting mechanical articulation movements (Freitas de Souza et al. 2007; Kikutani et al. 2009). Second, dentures alter planning, control and self‐monitoring of articulation movements by proprioception and sensation as they cover parts of sentient oral tissue (Ghi and McGivney 1979; Hermes et al. 2018). Third, if the participants had ill‐fitting or poorly adhered dentures, it may have caused them either to slow down or to make compensatory efforts in their speech movements (Grasso et al. 1994). Fourth, missing teeth may indicate weakened general health and function. Individuals of poorer health are known to speak slower (Duchin and Mysak 1987) and individuals with frailty have been noted to show slower DDK rates (Dos Santos Nogueira et al. 2023). For these reasons, the negative impact of dentures on the AMR tasks and narrative articulation rate is plausible. Our results conform to previous research in highlighting the significance of oral health to speech production (Hara et al. 2017; Kikutani et al. 2009).

Education was another significant demographic factor in this study. Educational level can be seen as a proxy for cognitive and intellectual skills, but it also has inevitable socio‐economical and psychological bearing (see Alantie et al. 2022). Bachelor's, Master's, or equivalent level of education predicted the slowest rates in all AMR tasks. As we found no previous studies on the topic, the typicality and cause of this finding can only be speculated. On one hand, it is possible that highly educated VO speakers use more cognitive effort than their less educated peers. Highly educated speakers might have a stronger metacognitive and metalinguistic awareness in the testing situation, which might hinder them from performing at maximal speed. They might, for example, try to maintain a steady pace and rhythm, try to mimic the researcher's exemplified pace or try to avoid any coarticulation for the sake of speech clarity. We are also aware of the possibility of mere coincidence or the influence of some uncontrolled demographic variable. According to Jacewicz et al. (2009), speech tempo is affected by a complex interaction of within‐speaker factors, of which education is but one. In addition to educational history, for example, personality, style or strategic choices could have affected any of the speech tasks conducted in this study.

Additionally, language skills were positively associated with motor–speech performance. Better phonemic fluency (k‐words per 60 s) predicted faster AMR /pa/ as well as SMR /pataka/. Phonemic fluency entails verbal, executive and memory skills (see Alantie et al. 2022). In the production of SMR /pataka/, all these abilities come into play, as a speaker needs to rapidly learn a new non‐word and retain it in the phonological loop, monitor its production in repetition and uphold a rapid production speed. The SMR task is both motorically and cognitively demanding, and it is not surprising that phonological fluency contributes to its execution. Why, then, is phonological fluency also associated with DDK AMR /pa/, but not with /ta/ or /ka/? We suggest both motorically and cognitively emphasised reasons. AMR /pa/ is the quickest and motorically easiest of the rates (Duffy 2013) and may, therefore, allow individual variations and predictor associations to occur more easily. It was also the first motor–speech task conducted in the testing procedure and was less likely than the rest of the DDK tasks to be affected, for example, by decreased attention or fatigue.

Higher WAB AQ was associated with faster narrative speaking rate. Speaking rate carries a considerable amount of information on motoric and language skills, as it incorporates the time used in articulatory movements for formulating words (Bóna 2014) with pauses signalling cognitive and communicative processing (Spieler and Griffin 2006). Articulation rate, which does not include pauses, was not statistically related to WAB AQ. This is why we conclude that pausing is especially affected by language skills in VO speakers. According to, for example, Spieler and Griffin (2006), pausing may signal word‐finding or speech‐processing difficulties. In this light, the significant connection between language proficiency scores, such as WAB AQ, and speaking rate is rational. Altogether, the finding provides us with further view on the relationship between the two generally utilised clinical assessment methods as well as between linguistic and motoric facets of speech production. We did not account for the frequency, mean length or placement of the pauses, which could be a sensible topic of future research.

The rest of the background variables, that is, hearing, MMSE and semantic fluency, did not have predictive power on the motor–speech tasks. Hearing did not reach statistical significance, perhaps because all the speakers could hear and comprehend the task instructions sufficiently well, and the task executions did not require fine‐tuned auditory self‐monitoring. MMSE had no association with the tasks, probably because the cut‐off for acceptance for this study was set at 24 points, which is the cut‐off score for typical cognitive function (Hänninen et al. 2010). Semantic fluency, again, did not predict motor–speech performance, as the two probably do not share enough common ground of cognitive‐linguistic processing requirements, unlike in the case of phonemic fluency.

### 4.4 Pragmatic Clinical Applications

We offer a few practical tips for utilizing the normative information provided by this study. We especially recommend testing all AMR /pa/, /ta/, /ka/ and SMR /pataka/ in clinical settings, as their atypical age‐related speed or speed order (in VO speakers SMR is typically slower than AMR) has the potential to indicate neuromotor or neurocognitive problems. To evaluate if a client performs within the normal range, their task performance can be compared to the two standard deviations of the task performance given in this study. An alternative way is to use a freely available program, for example, Singlims_ES.exe (Crawford 2025; Crawford and Garthwaite 2002; Crawford et al. 2010), which allows for easy statistical comparison of an individual speaker's performance to the reference data by using a modified *t*‐test (if the two‐tailed probability is < 0.05, the tested speakers’ performance deviates from the reference data).

### 4.5 The Limitations of the Study

While this study offers new insight, the following limitations should be acknowledged. Hearing was screened by mere self‐evaluation, which may not have been a completely reliable way to assess auditory competence. The inclusion of at least a basic hearing screening is relevant in future studies. In addition, this study did not include tests of non‐speech oral functions, although this would have allowed taking the exploration of the interplay between bodily and cognitive functions in ageing even further. In future studies, a real‐world repetition task should be included alongside the non‐word repetition (SMR). This could improve differential diagnosis of typical and pathological speech alteration, as real‐ and non‐word oral‐DDK rates may be differentially affected by age and distinct pathologies (see Ben‐David and Icht 2017). This study does not offer a comparison between neurologically healthy speakers and clinical groups, but it offers reference data for future studies involving growing populations of neuropathological and bi/multilingual speakers.

## 5 Conclusions

Regardless of their honourably high age, the very old (VO) speakers showed relatively well‐preserved motor–speech skills. However, like in earlier studies, the mean DDK, speaking and articulation rates were consistently slower in the VO speakers compared to prior Finnish data on adults and younger elderly. We found physio‐anatomical and cognitive‐linguistic predictors of motor–speech task performance in the VO speakers. Older age within the 80–100‐year range, wearing dentures (vs. natural teeth) and high educational level predicted slower rates in the motor–speech tasks. On the contrary, good language skills, namely better phonemic fluency and higher WAB AQ, were associated with faster rates. The results imply that oral DDK and narrative speech tempo are related to cognitive‐linguistic skills, potentially drawing at least in part on overlapping neural processes. The normative data of this study will help identify typical motor–speech slowing in VO speakers with varying background factors.

## Ethics Statement

Ethical approval for this study was obtained from the Regional Ethics Committee of the Tampere Human Sciences in 2018.

## Consent

Written informed consent was provided by all the participants. The volunteers did not receive any financial remuneration for participating.

## Conflicts of Interest

The authors declare no conflicts of interest.

## Data Availability

The dataset utilized in this article is not available for public access. For data sharing request please contact the first author.

## Appendix A Variable means, standard deviations, and correlations with confidence intervals

**Table:**

Variable	*M*	*SD*	1	2	3	4	5	6	7	8	9	10	11	12	13
**1. Age**	84.84	4.06													
**2. Education^a^**	1.88	0.77	−.10												
			[−.37, .19]												
**3. Hearing^b^**	1.50	0.51	.13	.10											
			[−.15, .39]	[−.18, .37]											
**4. Dentition^c^**	1.54	0.50	.22	−.30*	−.04										
			[−.06, .47]	[−.54, −.03]	[−.31, .24]										
**5. MMSE**	27.58	1.79	−.32*	.33*	−.01	−.06									
			[−.55, −.04]	[.06, .56]	[−.29, .27]	[−.33, .22]									
**6. WABAQ**	95.68	3.41	−.45**	.29*	−.02	−.30*	.39**								
			[−.65, −.19]	[.01, .53]	[−.29, .26]	[−.54, −.03]	[.13, .60]								
**7. SF**	21.16	6.80	−.39**	.42**	−.04	−.36*	.38**	.67**							
			[−.60, −.12]	[.15, .62]	[−.31, .25]	[−.58, −.09]	[.12, .60]	[.48, .80]							
**8. PF**	13.92	5.62	−.07	.37**	−.04	−.22	.42**	.46**	.58**						
			[−.34, .21]	[.11, .59]	[−.32, .24]	[−.47, .07]	[.16, .63]	[.21, .66]	[.35, .74]						
**9. /pa/**	6.17	0.62	.04	−.15	.02	−.28*	.08	.24	.18	.37**					
			[−.24, .32]	[−.41, .14]	[−.26, .30]	[−.52, −.00]	[−.21, .35]	[−.04, .49]	[−.11, .44]	[.09, .59]					
**10. /ta/**	6.02	0.68	−.11	−.15	.01	−.25	.09	.26	.07	.22	.76**				
			[−.38, .17]	[−.41, .13]	[−.27, .29]	[−.50, .03]	[−.19, .36]	[−.02, .50]	[−.21, .34]	[−.07, .47]	[.60, .86]				
**11. /ka/**	5.64	0.67	−.02	−.12	−.14	−.23	.01	.23	.11	.20	.68**	.77**			
			[−.30, .26]	[−.39, .16]	[−.41, .14]	[−.48, .05]	[−.27, .29]	[−.05, .48]	[−.17, .38]	[−.08, .45]	[.49, .81]	[.63, .87]			
**12. /pataka/**	5.52	0.93	−.26	.05	−.02	−.27	.32*	.38**	.37**	.44**	.53**	.50**	.34*		
			[−.50, .02]	[−.23, .33]	[−.30, .26]	[−.52, .01]	[.04, .55]	[.11, .60]	[.10, .59]	[.18, .64]	[.28, .70]	[.25, .68]	[.07, .57]		
**13. SR**	3.19	0.65	−.13	.24	−.12	−.29*	.26	.46**	.44**	.41**	.44**	.38**	.33*	.41**	
			[−.40, .15]	[−.05, .48]	[−.38, .17]	[−.53, −.02]	[−.02, .50]	[.20, .65]	[.18, .64]	[.14, .61]	[.18, .64]	[.12, .60]	[.06, .56]	[.14, .62]	
**14. AR**	4.81	0.67	−.19	−.04	−.21	−.30*	.12	.12	.05	.10	.43**	.51**	.40**	.45**	.58**
			[−.45, .09]	[−.31, .25]	[−.46, .08]	[−.53, −.02]	[−.16, .39]	[−.17, .38]	[−.23, .32]	[−.18, .37]	[.17, .63]	[.26, .69]	[.14, .61]	[.19, .65]	[.36, .74]

*Note. M* and *SD* are used to represent mean and standard deviation, respectively. Values in square brackets indicate the 95% confidence interval for each correlation. The confidence interval is a plausible range of population correlations that could have caused the sample correlation. ^a^ Primary level education as reference group; ^b^ Adequate hearing (vs. poor) as reference group; ^c^ Natural teeth (vs. dentures) as reference group. MMSE = Mini Mental Sate Examination; WABAQ = Western Aphasia Battery Aphasia quotient; SF = semantic fluency (animal names per minute); PF = phonemic fluency (k‐words per minute); /pa/ = alternating motion rate monosyllable /pa/ in syllables per second; /ta/ = alternating motion rate monosyllable /ta/ in syllables per second; /ka/ = alternating motion rate monosyllable /ka/ in syllables per second; /pataka/ = number sequential motion rate trisyllable /pataka/ in syllables per second; SR = speaking rate in syllables per second; AR = articulation rate in syllables per second

## Appendix B Detailed description of the language variables

### Western Aphasia Battery – The Finnish version

The Finnish version of the WAB (Kertesz et al., 2005) is standardized and validated with altogether 314 Finnish speakers with and without aphasia (speakers without aphasia: *n* = 179, age range = 19–79, WAB‐AQ *M* = 98.1). To assess general language performance, we conducted the linguistic tasks and calculated aphasia quotient (WAB‐AQ, max 100) for this study. The first two authors conducted a consensus analysis on a randomly selected 30% of the WAB spontaneous speech samples to ensure interrater reliability, as the subtask is complex to score and requires clinical expertise.

### Semantic fluency

For semantic fluency, the participants were asked to produce as many animals as they could think of within 60 seconds. Semantic fluency was scored by the total number of correct and unique animal names produced within the minute. The following scoring principles were applied: Age (Pekkala, 2004; Pekkala et al., 2009) or gender variations (Kavé, 2007; Pekkala, 2004; Pekkala et al., 2009) of the same species (e.g.,“hevonen”/“horse,” “varsa”/“foal,” and “tamma”/“mare”) were counted as one item. Words presenting different species were counted as separate items (e.g., “talitintti”/“great tit,” “varpunen”/“sparrow,” “pöllö”/“owl”). If the names of specific species were accompanied with a general superordinate word (e.g., “lintu” or “linnut”/“bird” or “birds”), the superordinate was not scored, but if a superordinate word occurred alone, it was counted as a correct word (e.g., “lintu”/ “bird,” “kissa”/“cat,” “koira”/“dog”; see also, e.g., Kavé, 2007). Repetitions, neologisms, and outside category intrusions were discarded. There is normative reference data for Finnish adults and elderly on animal fluency with the same counting protocol as in this study (Pekkala et al. 2009: *n* = 30, *M* age = 66.7, *M* animals = 18.9/min). There is also normative reference data on adult and elderly Finnish speakers with a slightly different counting protocol (Lehtinen et al. 2023: *N* = 50, *M* age = 62.6, *M* animals = 26/min).

### Phonemic fluency

For the phonemic fluency task, we chose the letter k for its high frequency in the Finnish language. The participants were instructed to generate as many words as possible beginning with the letter k within 60 seconds and to avoid proper nouns. Compound words with the same first part as well as affixed words were counted as separate items providing that the words entailed separate meanings (e.g., “kukkavaasi”/flower vase vs. “kukkakauppa”/flower shop, “kana”/hen vs. “kanala”/henhouse). Repetitions, proper nouns, neologisms, and words beginning with a wrong phoneme were excluded. To ensure counting and scoring accuracy, the two first authors independently analyzed a randomly chosen sample of 30% of both the semantic and phonemic category fluency tasks to form a consensus analysis. There are standardized and validated norms (Manninen et al., 2015: *N* = 62, age range: 18–65, *M* letter‐k fluency = 19.2 k‐words/min) and reference data (Lehtinen et al., 2023: *N* = 50, *M* age = 62.6, *M* k words = 19.4/min) for phonemic fluency in adult Finnish speakers.
